# Genomic prediction accuracy for low hydrogen cyanide selection in fresh cassava roots. Comparative model analysis

**DOI:** 10.1186/s12870-026-08650-3

**Published:** 2026-03-31

**Authors:** Michael Kanaabi, Settumba B. Mukasa, Ephraim Nuwamanya, Ismael Siraj Kayondo, Henry Wagaba, Paula Iragaba, Julius K. Baguma, Nicholas Muhumuza, Ann Ritah Nanyonjo, Enoch Wembabazi, Mary Buttibwa, Williams Esuma, Robert Kawuki

**Affiliations:** 1 College of Agricultural and Environmental Sciences, Makerere University, P.O. Box 7062, Kampala, Uganda; 2 National Coffee Research Institute (NaCORI), P.O. Box 185, Mukono, Uganda; 3 International Institute for Tropical Agriculture (IITA), Ibadan, 200113 Nigeria; 4 National Agricultural Research Laboratories (NaRL), P.O Box 7065, Kampala, Uganda; 5 National Crops Resources Research Institute (NaCRRI), P.O. Box 7084, Kampala, Uganda; 6 World Coffee Research (WCR), 1094 SW Barnes Road #334, Portland, OR 97225 USA

**Keywords:** Food safety, Genetic gain, Genomic selection, Hydrogen cyanide, Marker assisted selection

## Abstract

Cassava (*Manihot esculenta* Crunz) is a staple food crop for millions of people in Africa. The crop’s tolerance to drought, ability to survive in marginal soils and amenability to a variety of uses endears it to farmers. However, utilization of the crop’s full potential for food security is limited by the presence of cyanogenic glucosides (HCN) in the roots. These not only make cassava bitter to the taste but can also be toxic to lethal levels. Breeding for low HCN cassava is necessary to mitigate against high dietary HCN consumption. However, efforts at variety development have been slow, in part due to limited use of molecular tools. This study assessed genomic prediction accuracy for fresh cassava root HCN content using the GBLUP model and compared its performance to 5 Bayesian models (Bayes A, Bayes B, Bayes C, Bayesian Ridge Regression (BRR), Bayesian Lasso (BL) and the Reproducing Kernel Hilbert Spaces (RKHS) model. We used a genomic selection (GS) cycle two population consisting of 434 clones which were genotyped with 24,040 SNP markers using the DArTseq platform. Data were filtered to remove markers with minor allele frequency (MAF) less than 0.05, call rate less than 80%, clones with more than 20% missing calls and clones with no matching phenotypic data. Phenotypic data on fresh cassava root HCN content was collected on roots at 12 months after planting. The accuracy of GBLUP model was low, ranging from -0.41 to 0.68 and averaging 0.22 across folds. On the other hand, the accuracy of RKHS and Bayesian models was moderate, with BL and RKHS being most accurate (*r =* 0.52), followed by Bayes A (*r =* 0.49) and Bayes B and Bayes C (*r =* 0.48). The BRR model was least accurate (*r =* 0.18). Collectively, these results provide a foundation for implementing GS to accelerate efforts to deliver desired genetic gains for low HCN cassava in farmers’ fields.

## Background

Cassava is a staple food crop for millions of people in Africa, Asia and South America. In addition to having multiple uses for food, feed and industry, the crop is hardy and survives well in marginal soils and under drought conditions [1, 2], making is a major food security crop for resource limited farmers in the face of changing climatic conditions. However, the usefulness of the crop is limited by the presence of the cyanogenic glucosides linamarin and lotaustralin in the leaves and roots. In neutral or alkaline media and in presence linamarase enzyme, linamarin readily hydrolyses into glucose and acetone cyanohydrin. The acetone cyanohydrin decomposes to liberate cyanide and a hydrogen ion (HCN) [3, 4]. In the cassava plant, linamarin and lotaustralin are stored in the cell vacuoles while linamarase is stored in the cell wall. This spatial separation ensures that cyanide release does not occur in intact tissues. However, when root tissues are damaged say by peeling, grating or chewing, cell structures are broken down, allowing interaction of linamarin with linamarase, leading to liberation of volatile hydrogen cyanide (HCN) [3]. This HCN makes the cassava roots bitter [5]. Chronic dietary exposure to HCN is associated with the neurological disorders; konzo [6, 7] and tropical ataxic neuropathy [8, 9] while ingestion of high HCN cassava can be acutely toxic [10, 11]. Whereas communities traditionally cultivating high HCN cassava varieties know processing techniques for HCN reduction, sometimes the processing is not effective [12] and when these roots are sold in distant markets, buyers may not always be aware of the potentially lethal levels of HCN in the roots [10, 11]. The most sustainable way to guard the public from harmful effects of dietary HCN consumption is by breeding for low HCN cassava varieties that meet other user preferences. However, breeding efforts have been slow due to challenges associated with HCN phenotyping as current methods [13–16] are slow, laborious and/or limited in accuracy.

A significant challenge in cassava breeding is the crop's long cycle time, which can take up to 12 months to reach physiological maturity. For traits such as fresh cassava root HCN content, which are evaluated at physiological maturity, it can take a full year to advance from one breeding stage to the next. Moreover, the ability to select individuals at early breeding stages is crucial for maximizing genetic gains and accelerating the development of new varieties, making it a key objective for cassava breeding programs [17]. Genomic selection (GS) leverages genome-wide markers to predict an individual's genetic merit, enabling selection decisions to be made earlier [18, 19]. The availability of genome-wide markers makes GS applicable for traits controlled by a few major genes like the oligogenic HCN [20] as well as those influenced by many small-effect genes. Compared to traditional phenotypic selection, GS allows for selection before phenotyping, reducing costs and, in some cases, proving more accurate than marker-assisted selection [21, 22]. Whereas the potential of deploying marker assisted selection for low HCN cassava breeding has been variously demonstrated [23–26], only one published study [25] evaluated the possibility of genome-wide markers assisted selection for HCN to facilitate germplasm exchange between breeding programs.

Historically, the Uganda cassava breeding program has employed genomic selection for the cyclic improvement of traits such as resistance cassava brown streak disease [19, 27, 28], dry matter content, and total carotenoids [18]. Prediction accuracies, achieved using the genomic best linear unbiased predictor (GBLUP) and Bayesian models, have ranged from −0.16 to 0.68 [18, 19, 27, 28]. The GBLUP model utilizes a genomic relationship matrix (GRM) built from SNP marker data to capture the genetic relationships between individuals. It applies a linear mixed model where the genetic effects, represented by the GRM, are treated as random effects [29]. The inverse of the genomic relationship matrix was calculated using the *G.inverse* function and used to account for population structure and relatedness. The model then predicts the genetic value of individuals by aggregating the effects of individual markers across the genome. The GRM enables more precise estimation of genetic relationships, resulting in improved predictions [30]. Additionally, it accounts for linkage decay, which refers to the non-random association of alleles at different loci, thereby enhancing prediction accuracy. Bayes A model assumes that marker effects follow a t-a prior distribution and shrinks the estimates of marker effects towards zero, thereby reducing the risk of overfitting. Marker effects are assumed to be independent of each other, and a few markers are assumed to have large effects while many others have small effects [31]. Similar to Bayes A, Bayes B assumes that marker effects are independent and that most markers have no influence on the trait. The markers that influence the trait are assumed to follow a scaled-t prior distribution [32]. In contrast, Bayes C operates under the assumption that only a small proportion of SNPs have moderate to large effects, while the majority have no effect. Additionally, Bayes C integrates prior knowledge about the distribution of SNP effects [32] In contrast, Bayesian Ridge Regression (BRR) assigns a Gaussian prior with a shared variance to each marker effect, applying uniform shrinkage across all marker effects [31]. On the other hand, the Bayesian Lasso (BL) model assumes that SNP effects are independent and uses a double-exponential prior distribution for marker effects, which heavily shrinks markers with little or no impact on the trait [33, 34]. The primary differences between the models lie in their assumptions regarding marker effects.

In Reproducing Kernel Hilbert Spaces (RKHS), a Gaussian kernel serves as a basis function to estimate conditional expectations. The goal is to reduce numerous genetic markers into a positive semi-definite matrix of size n x n, where n represents the number of phenotypes (HCN scores), by constructing genetic kinship based on spatial distance within a specific metric space. For this study, the Gaussian kernel was utilized.

$${K}_{ij}=\mathrm{exp}(-\left({d}_{ij}\uptheta \right))$$

Where ${K}_{ij}$ is the computed relationship between two cloness, ${d}_{ij}$ is the Eucladian genetic distance based on marker dosages and Ɵ is a tuning parameter that determines the rate of decay of correlation among clones.

The performance of the GBLUP model has been comparable to or better than that of the Bayesian models. However, there is no universally optimal genomic prediction model, as a set of assumptions that work well in one scenario may not perform effectively in another [35]. The accuracy of genomic predictions can be affected by the genetic architecture of the trait, the trait heritability, marker density, size of the training set, population structure, genetic variance and choice of genomic prediction model among others [27, 28, 36–38]. Therefore, it is essential to evaluate multiple models to determine which one is best suited for the HCN dataset.

Whereas this genomic selection cyclic population has been studied for cassava mosaic and cassava brown streak disease no similar work has been done for root quality traits, despite root quality being a major consideration in selection of parental genotypes in the genomic selection cycle zero (C0) population. The phenotyping of cassava root HCN content is complex given that it is costly, slow and highly labor-intensive. For example, using the picrate paper method [39], one has to prepare picrate paper strips which require at least 24 h of air drying after dipping them in picrate solution (solution of picric acid and sodium carbonate), dig roots out of the ground, wash to remove debris, peel to remove the cortex, weigh off a sample for the analysis, expose the sample to the picrate paper strip, incubate in the dark and wait for 12 to 16 h before matching the color change on the paper strip to a color chart. This difficulty in HCN phenotyping coupled with the fact that the trait is only evaluated after physiological maturity, makes optimizing genomic predictions for HCN a high priority as it would accelerate genetic gains for low HCN cassava breeding while significantly reducing the cost of selecting low HCN cassava accessions. Besides, information on genomic predictions for HCN in fresh cassava roots is limited. Accordingly, this study aimed at assessing the possibility of using GS as a breeding tool for selection of accessions with low HCN, an intricate and yet desirable quality trait. This possibility was done through undertaking two empirical studies to; 1) assess genomic prediction accuracy for fresh cassava root HCN content using the GBLUP model 2) compare performance of the GBLUP model to 5 Bayesian models (Bayes A, Bayes B, Bayes C, Bayesian Ridge Regression (BRR), Bayesian Lasso (BL) and the Reproducing Kernel Hilbert Spaces (RKHS) model. It is justified to evaluate several models since no single model is best fit for all data sets [31, 40]. Moreover, different genomic prediction models impose different computational demands on the breeding program. Thus, for resource limited publicly funded breeding programs, identifying models that are computationally efficient improves their efficiency given that GS often involves thousands of individuals and thousands of markers. Moreover, increasing model complexity can incur huge computational costs without yielding proportional gains in predictive accuracy [41].

## Materials and methods

### The study population

The study material consisted of a genomic selection cycle two (C2) clonal population [27]. This population originated from a cycle zero (C0) genomic selection population, which included 348 clones derived from forty-nine progenitors obtained from the National Crops Resources Research Institute (NaCRRI), the International Institute of Tropical Agriculture (IITA), and the International Centre for Tropical Agriculture (CIAT) in 2013. Progenitors from CIAT were selected based on their root quality performance, while those from NaCRRI and IITA were chosen for their fresh root yield, resistance to cassava brown streak disease (CBSD), and root quality traits. Elite progenitors from this group were identified using their genomic estimated breeding values (GEBVs) and used to develop a cycle one (C1) population comprising 638 clones. Subsequently, elite progenitors from C1 were crossbred to produce 6,570 half-sib and full-sib seedlings, forming the genomic selection cycle two (C2) population. These seedlings were established in a field at NaCRRI in 2018 to multiply planting material.

**Fig. 1:**
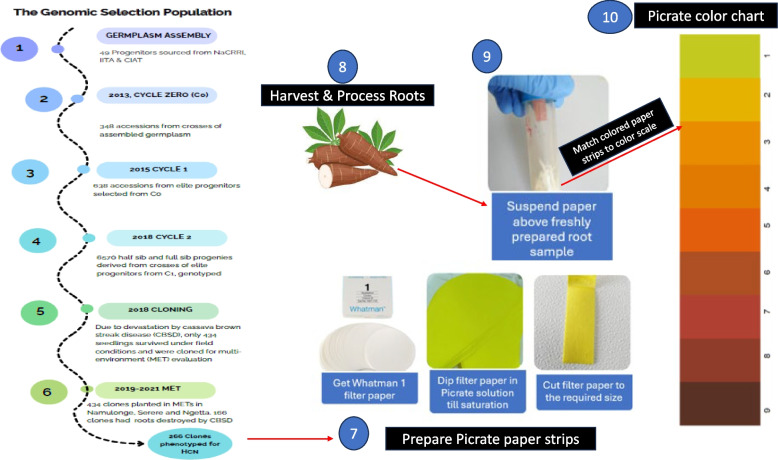
Workflow for generating the cassava genomic selection population and phenotyping cassava roots for HCN using the Picrate paper color scale protocol

Due to devastation by cassava brown streak disease, only 434 seedlings survived with sufficient planting material to establish multi-location trials (Fig. 1). These were cloned and established in field trials in Namulonge (2019, 2020), Serere (2019, 2020, 2021) and Ngetta (2021) in an augmented design [18] consisting of 29 blocks with 2 checks per block. Each block had 15 plots (accessions), with each plot consisting of a single row of 10 plants. The plants were established at a spacing of one meter between plants in a row and one meter between adjacent rows. Routine weeding was done using a hand-hoe to maintain the fields weed free. In the end, phenotypic data was collected from only 266 clones since we could not obtain sufficient roots from 168 clones owing to damage by cassava brown streak disease necrosis, which rendered them unsuitable for evaluation for HCN content [42].

### Sampling for genotyping, DArTseq genotyping and SNP quality control

Leaf sampling for genotyping was done three months after planting in the field trial established in at NaCCRI in Namulonge. Two young fully opened leaves were identified and four-leaf punches collected form these using a 5 mm cork borer. Fresh leaves were placed in a 96-well genotyping plate, dried using silica gel in a desiccator. DNA extraction, genotyping, and SNP calling were performed using the DArTSeq platform (https://www.diversityarrays.com/services/dartreseq/). The resultant sequences were aligned to cassava reference genome version 6.1. A total of 240,40 markers were called. The SNPs were then filtered to remove those with minor allele frequency less than 0.05, call rate less than 80% and more than 20% missing calls using the *qc.filtering* function in *ASRgenomics* R package [43]. After filtering, 4594 SNPs with MAF less than 0.05 and 10 clones with more than 20% missing calls were removed, leaving 19,446 markers and 255 clones that were used for downstream analysis.

### Phenotypic data collection

At maturity, 12 months after planting, the roots of the five middle plants were harvested and combined. Three healthy (non-necrotic), uniformly sized roots were selected and transported to the laboratory. The roots were rinsed under running water to remove soil and debris, then dried with a kitchen towel. The extreme ends (distal and proximal ends) of each root were cut off and discarded, leaving a 5 cm middle section. This middle portion was peeled and grated using a kitchen grater. The grated material from each root was wrapped together, and a sample was immediately weighed into a falcon tube containing 5 mL of pH 8 phosphate buffer, with duplicates prepared for each sample, making 6 observations per plot. A fresh picrate paper strip (1 cm wide, 3 cm long), prepared by dipping Whatman 1 filter paper in a yellow solution of picric acid (0.5% w/v) and sodium carbonate (2.5% w/v) and allowing the paper to dry for 24 h at room temperature was suspended above the falcon tube, and the tube was tightly sealed. The tubes were incubated in the dark for 16 h at room temperature and scored on a scale of 1 to 9, where 1 and 9 represent extremes of low and high HCN respectively (Fig. 1) [39]. Because HCN phenotyping is labor intensive and time consuming, only 20 randomly selected plots were processed per day. The 6 observations per plot were averaged to get a mean plot HCN score which was used for downstream analysis.

### Data analysis

#### Phenotypic data analysis

The data collected over various locations and years was combined into a single file. Histograms were created using the *ggplot2* package in R to visualize the data distribution. A mixed linear model was then fitted using the *ASReml-R* package in R, which estimated variance components via the restricted maximum likelihood method [44]. These variance components were subsequently used to calculate broad-sense heritability (H^2^). The linear mixed model fitted was as below:

$$\begin{aligned}y&={\rm X} \beta +Z{clone}^{c}+Z{{block}^{b}}_{location}\\&+Z{{clone}^{l}}_{location}+\varepsilon\end{aligned}$$

Where y represents the HCN phenotype, $\beta$ is the fixed effect for the population mean with ${\rm X}$ the corresponding incidence matrix. The incidence matrix ${Z}_{clone}$ and vector c represent fixed effects of clones where $\mathrm{C}\sim N(0, {\boldsymbol{I}}{\sigma }^{2}c$) with **I** being the identity matrix. The block nested within location effects were modeled as a random term with incidence matrix ${Z}_{block}$ and effects vector b ~ N (0, ${\boldsymbol{I}}{\sigma }^{2}b$). Vector l and incidence matrix ($Z{{clone}^{l}}_{location}$) represent the random effect of clone by location interaction and $\varepsilon$ term represents the residual, distributed as $\varepsilon \sim N(0, {\sigma }^{2}\varepsilon$).

Broad-sense heritability (H^2^) estimates were computed using the formula:

$${H}^{2}=\frac{{{\sigma }^{2}}_{c}}{{{\sigma }^{2}}_{c}+{{\sigma }^{2}}_{cl}+{{\sigma }^{2}}_{e}}$$

Where; ${{\sigma }^{2}}_{c}$ represents the variance attributed to the clone, ${{\sigma }^{2}}_{cl}$ denotes the variance due to the clone by location interaction, ${{\sigma }^{2}}_{e}$ is the residual variance. Narrow-sense heritability (h^2^) was calculated from the genomic relationship matrix via the formula:

$${h}^{2}=\frac{{{\sigma }^{2}}_{a}}{{{\sigma }^{2}}_{a}+{{\sigma }^{2}}_{e}}$$

Where; ${{\sigma }^{2}}_{a}$ represents the additive genetic variance, ${{\sigma }^{2}}_{e}$ denotes the non-additive genetic variance. The model was used to obtain Best Unbiased Linear Estimates (BLUEs) of each clone.

#### Assessment of population structure and kinship

After filtering genotype data and removing clones with more that 20% missing calls, 19,446 SNPs and 255 clones were retained and used for downstream analyses. The SNPs were formatted as a dosage matrix with genotypes coded as 0, 1 or 2 which represented alternative allele homozygotes, heterozygotes and reference allele homozygotes respectively using the *snp.recode* function in *ASRgenomics* R package. A kinship matrix was generated from a genomic relationship matrix (G) as described by Van Raden [29]. Let M be a marker matrix specifying marker alleles that each individual inherited. The dimensions of M are given by the number of individuals (*n*) by the number of loci (*m*). The genetic relationships between markers were evaluated using an *n* x* n* matrix MM´. The genomic relationship matrix G was then calculated from the formula,

$$G=\frac{ZZ{\prime}}{2{\sum }_{{P}_{i}}(1-{P}_{i})}$$

Where Z; is the difference between marker matrix M and the allele frequency (P). The allele frequency was expressed as the difference from 0.5 multiplied by 2 such that column *i* of P is 2($${P}_{i}-0.5)$$.

The kinship matrix was analyzed using the *kinship.diagnostics* function, and a plot of the off-diagonal values was generated using the *plot.offdiag* function. The diagonal elements of MM´ represent the inbreeding coefficient for each individual, while the off-diagonal elements measure the number of alleles shared among relatives (genetic relatedness among pairs of individuals). A heatmap of the genomic relationship matrix was plotted to visualize the population structure using the *kinship.heatmap* function. Additionally, principal component analysis (PCA) was conducted using the *kinship.pca* function from the *ASRgenomics* R package, and the first two principal components were plotted to further illustrate the population structure.

#### Genomic prediction models

The BLUEs obtained from phenotype data were used as a response variable to perform genomic prediction. The GBLUP model was implemented using the licenced software *ASReml-R* and the estimated breeding values were extracted using the *predict* function [45]. We fitted the model;

$$Y=1\beta +{X}_{g}+ \varepsilon$$ with $$g \sim N(0, K{\sigma }_{g}^{2})$$ and $$\varepsilon \sim (0, I{\sigma }_{g}^{2})$$

Where; Y is a vector of BLUEs, β is the overall population mean, X is a design matrix linking observations to genomic values, $g$ is a vector of genomic estimated breeding values for each clone and ε is a vector of residuals. We made the assumption that $g$ had a known covariance structure, defined by the realized genomic relationship matrix $K$ and identity matrix I.

Predictions using RKHS and Bayesian models were conducted with the BGLR package [46] in R. For the Bayesian methods, the same model as GBLUP was applied, assuming that the vector of estimated breeding values for each clone $(g)$ has an established covariance structure defined by the realized genomic relationship matrix and the identity matrix. The Bayesian methods were implemented using Markov Chain Monte Carlo (MCMC) sampling, incorporating the respective prior distributions for marker effects. For model tuning, the *burnIn* hyperparameter, which specifies the number of iterations at the beginning of the MCM chain that were ignored to remove bias from initial values was set to 700, the *nIter* hyperparameter which controls the total number of iterations of the MCMC sampler was set to 6000, the degrees of freedom (*df0*) were set to 5 while the scale parameter (*S0*) was set to 500.

#### Cross validation

A k-fold cross-validation approach was employed to assess the model's performance. The population was randomly divided into five subsets, and this process was repeated five times. During each cross-validation iteration, four of the five subsets were used as the training set, while the remaining one-fifth served as the validation set for model testing. Narrow-sense heritability was estimated separately within each validation set by portioning the phenotypic variance into genetic and error term components. The variance components were sampled from scaled inverse-chi square priors via MCMC. For each fold, posterior samples obtained after burn-in were averaged to yield the mean narrow-sense heritability for that fold. Genomic prediction accuracy was recorded for each iteration, and the process was repeated until every subset had been used as the test set, resulting into a total of 25 correlations. The model's predictive ability (*r*_*a*_) was evaluated based on the correlation between the BLUEs and the predicted values. Accuracy, on the other hand, was calculated by dividing the predictive ability (*r*_*a*_) by the square root of the narrow-sense heritability of the validation set [47, 48].

## Results

### Phenotypic variability

**Fig. 2:**
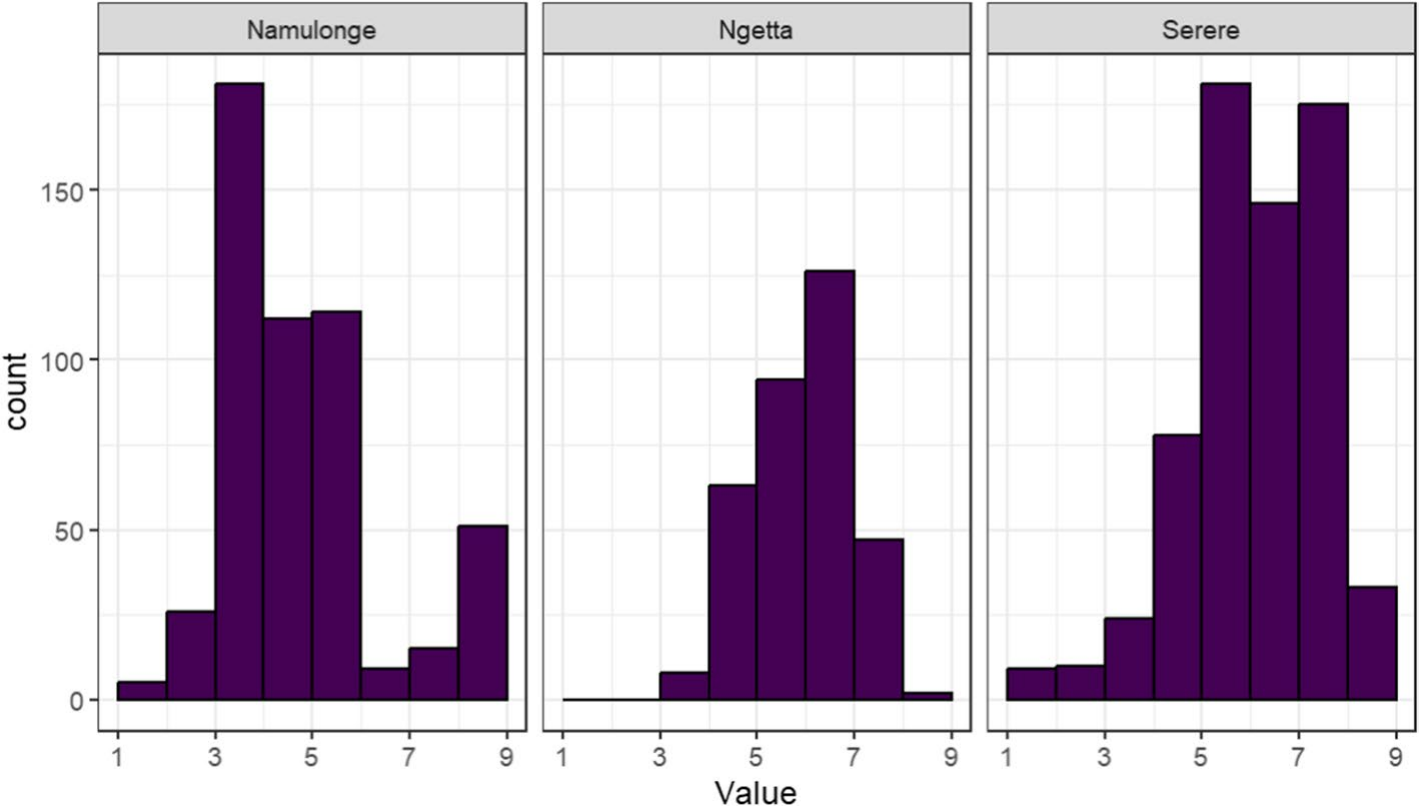
Distribution of fresh cassava root HCN content across study locations

The HCN phenotypes across locations and years were scored on a 1 to 9 scale. In Namulonge, the HCN phenotypic scores ranged from 1 to 9, averaging 5.3. In Ngetta, the scores ranged from 4 to 9, with an average of 6.4 while in Serere, HCN scores ranged from 1 to 9, averaging 6.6. Whereas HCN scores in Namulonge approximated a normal distribution, scores in Serere and Ngetta were skewed towards the high values (Fig. 2). The overall mean score across locations was 6.1. The broad-sense heritability (H^2^) across locations was 0.42. Overall, genomic selection is appropriate as the trait displays phenotypic variability.

### Population structure and kinship

**Fig. 3:**
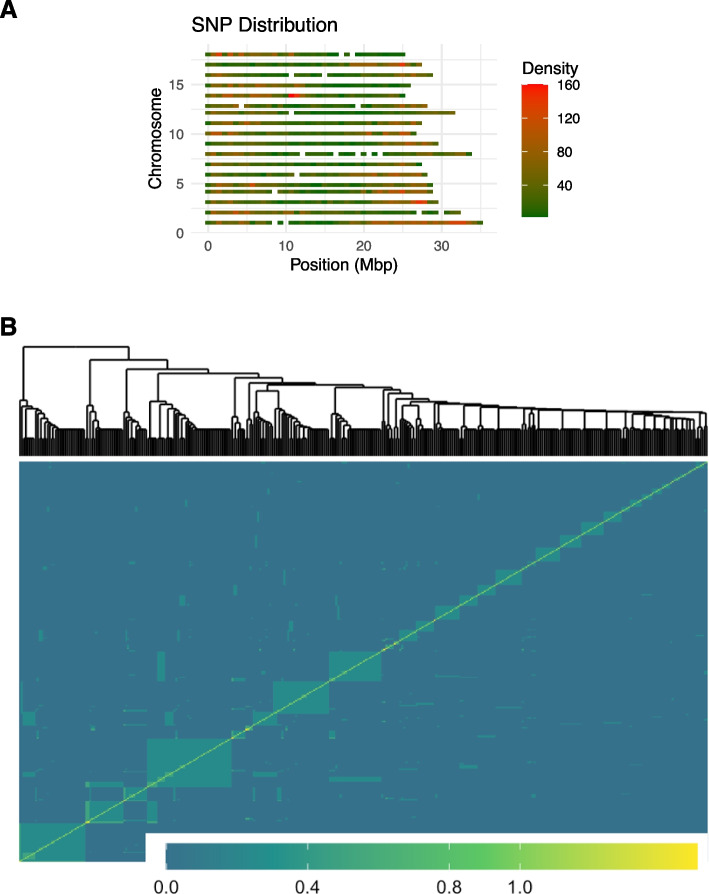
Distribution of SNPs on chromosomes (**A**) and heatmap of pairwise genomic relationship matrix based on 19,446 SNPs (**B**)

The SNP markers were distributed across all 18 chromosomes of the cassava genome (Fig. 3A). Population structure was investigated using a genomic relationship matrix and principal component analysis (PCA). A plot of genomic relationship matrix revealed kinship (relatedness) among some of the clones. Whereas the diagonal of the plot represents the kinship coefficient of an individual with itself, the off diagonal represents the kinship coefficient between different individuals in the population. The strength of the genetic relationship between individuals is signified by the color intensity from blue (no kinship) to yellow (full siblings) or parent-off spring relationships (Fig. 3B).

**Fig. 4:**
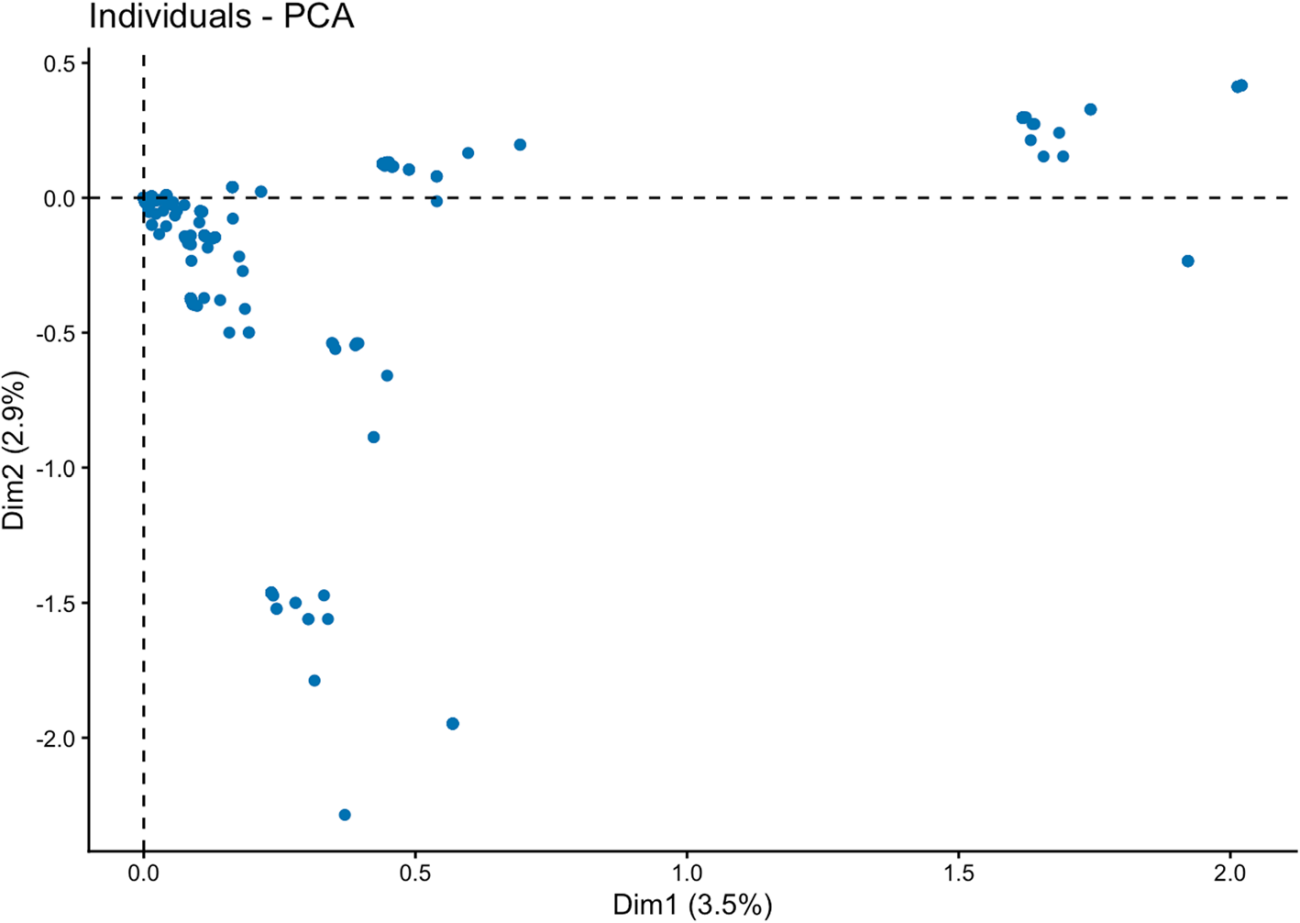
Principal component analysis biplot derived from the genomic relationship matrix

A biplot of the first two dimensions (principal components) generated from the genomic relationship matrix revealed that they accounted for 3.5% and 2.9% of the variation in SNPS. The first ten principal components accounted for 20.2% of the variation. Clustering is visible on the PCA bi-plot, implying presence of groups of individuals that are closely genetically related within the clusters but genetically distant from others in other clusters (Fig. 4).

### Cross validation accuracy and predictive ability for the GBLUP model

**Fig. 5:**
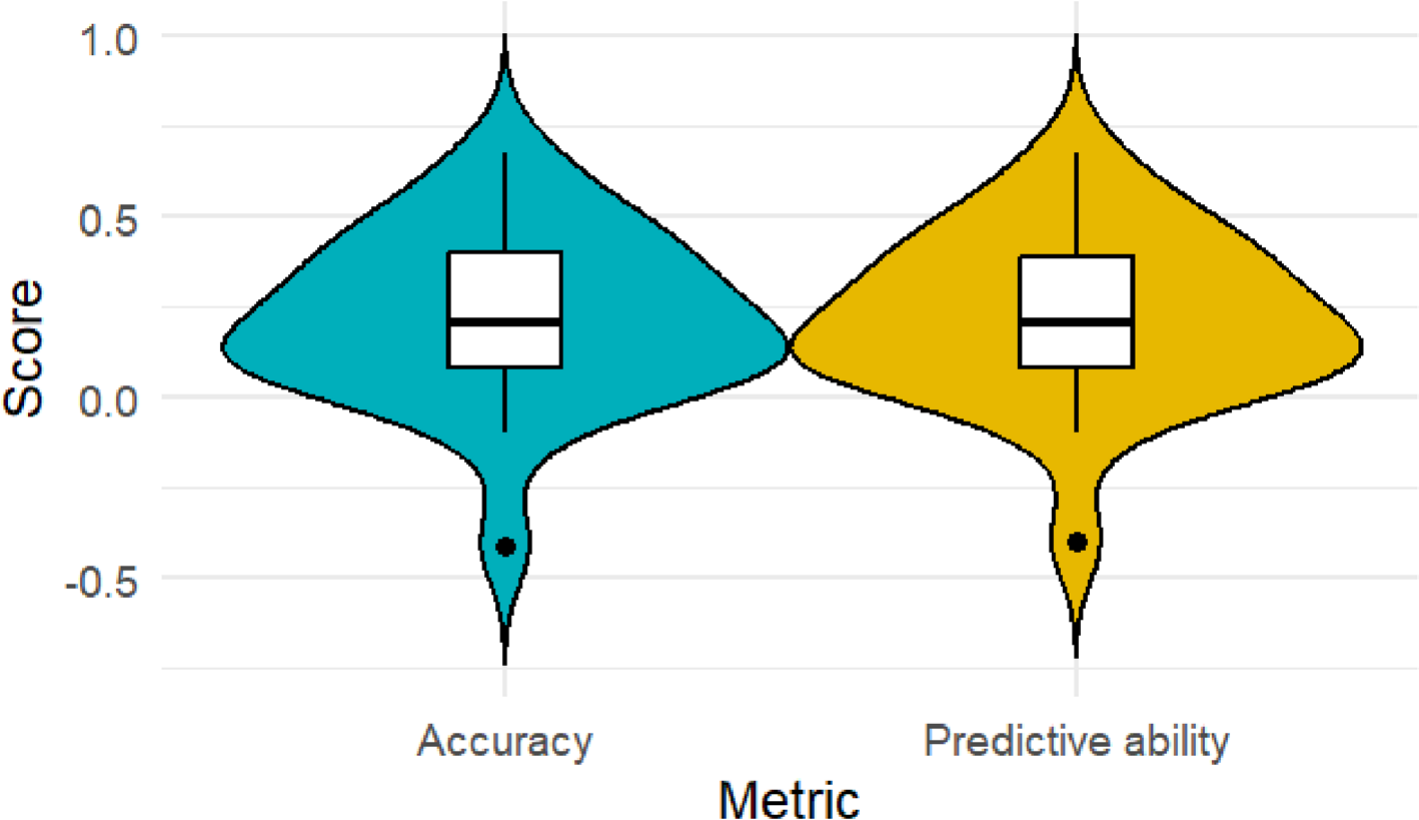
Variability in accuracy and predictive ability for the GBLUP model

**Fig. 6:**
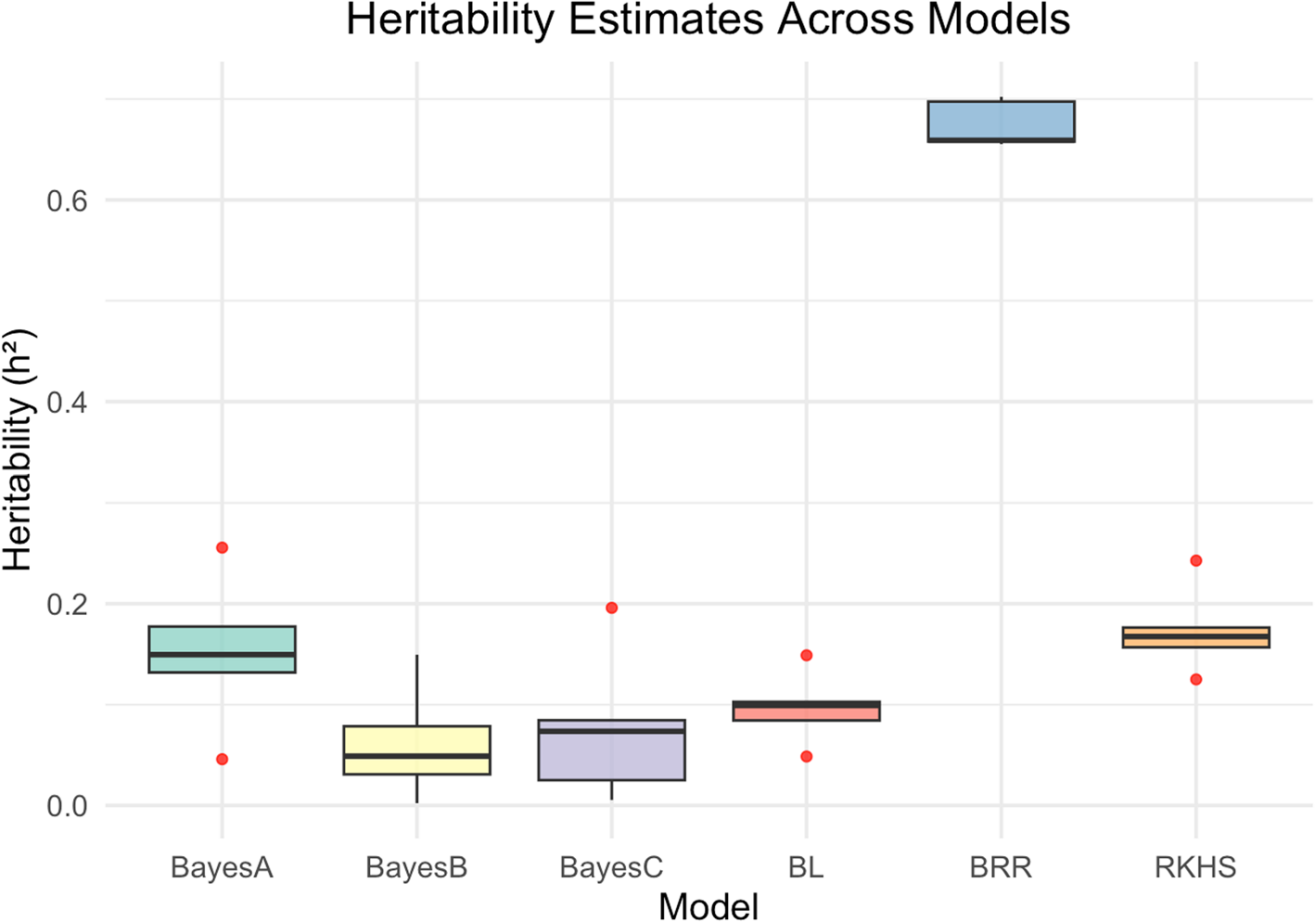
Box plots showing variability in narrow-sense heritability (h2) between the Bayesian models and the RKHS model

The iterations 1, 2 and 4 had positive values for accuracy (0.02–0.58) and predictive ability (0.02 −0.57). On the hand, folds 3 and 5 had variable prediction accuracy and prediction ability values, ranging from negative to positive, with the highest variability being recorded in fold 5 where accuracy ranged from −0.41 to 0.68 while predictive ability ranged from −0.4 to 0.68 (Fig. 6). In general, prediction accuracy was low to moderate across folds, ranging from −0.41 to 0.68 with a mean of 0.22. An identical trend was recorded for the predictive ability across iterations (mean = 0.22) (Fig. 5). The narrow-sense heritability (h^2^) was 0.35.

The accuracy of the GBLUP model ranged from −0.41 to 0.48 with an average of 0.22. The predictive ability of the model on the other hand ranged from −0.4 to 0.68, with a mean of 0.2 (Fig. 5). Across iterations, increasing the accuracy increased the predictive ability of the model. The narrow-sense heritability (h^2^) was 0.35.

### Performance of Bayesian and RKHS models

#### Narrow-sense heritability (h^2^) estimates for the Bayesian models and RKHS model

With the exception of BRR, the narrow-sense heritability estimates were low to moderate for the Bayesian and RKHS models across all folds. For Bayes A, h^2^ ranged from 0.05 to 0.30, while for Bayes B, it ranged from 0.002 to 0.15. Bayes C showed a range of 0.01 to 0.20, BL ranged from 0.05 to 0.15, and RKHS ranged from 0.13 to 0.18. The highest heritability per fold (0.66 to 0.70) was observed with the BRR model (Fig. 6).

#### Accuracy of RKHS and Bayesian models

**Fig. 7:**
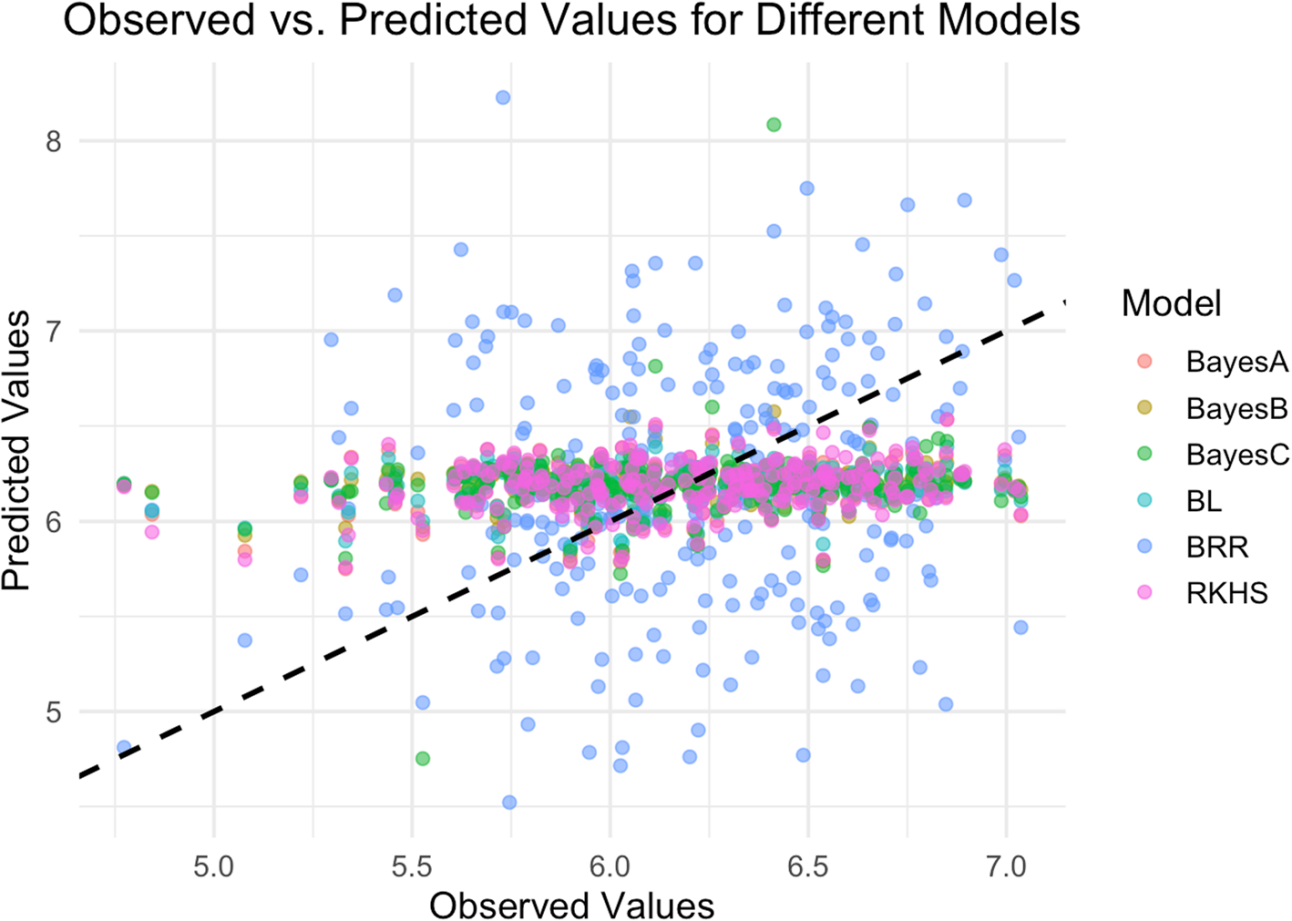
Scatter plots of observed (actual) HCN phenotype against the predicted HCN phenotype (BLUEs). The black line is the 1:1 line, a reference line where predicted values equal observed values for each model

With the exception of BRR, the accuracy of the models was moderate, ranging from 0.48 to 0.52. The Reproducing Kernel Hilbert Spaces and Bayesian Lasso models predicted better (*r =* 0.52), followed by the Bayes A model (*r =* 0.49) and Bayes B and C models (*r =* 0.48). The Bayesian Ridge Regression (BRR) model had a low accuracy (*r =* 0.18) (Fig. 7).

#### Cross validation predictive ability of RKHS and Bayesian models

**Fig. 8:**
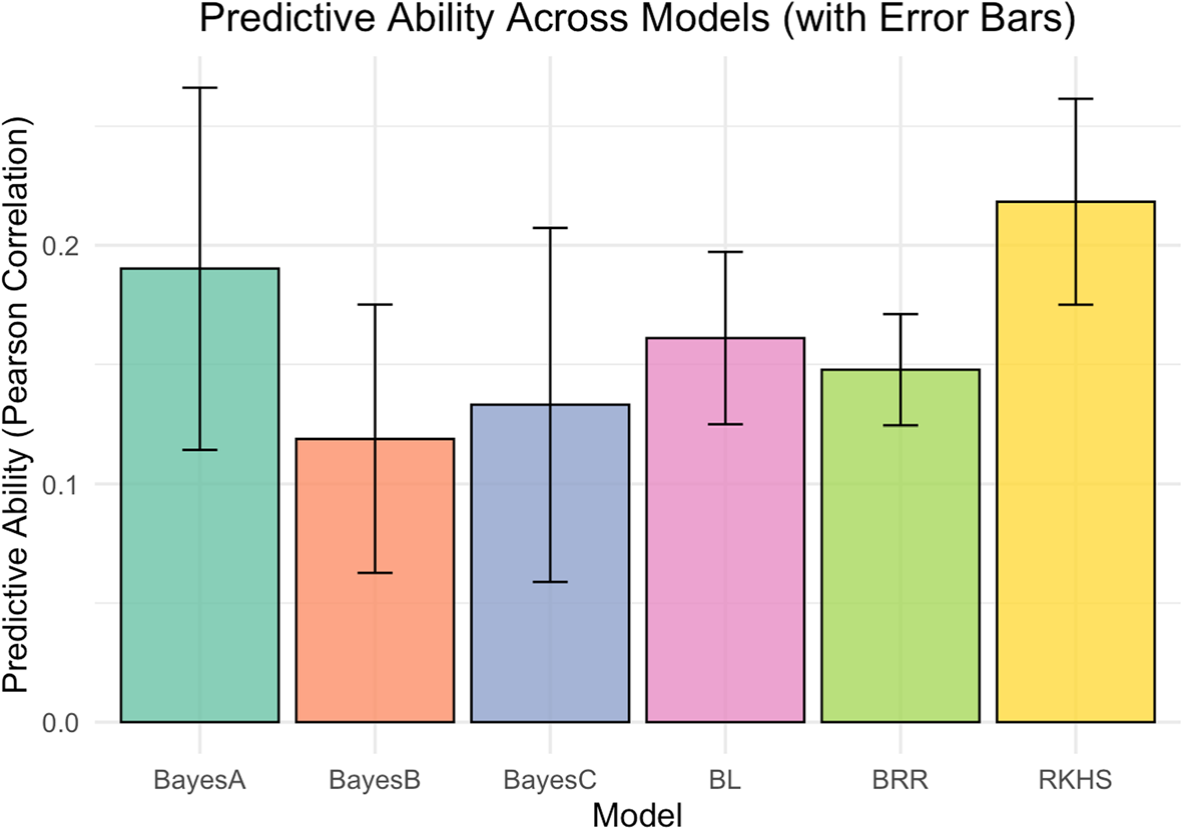
Variability in predictive ability across Bayesian models and RKHS model

Generally, the predictive ability of the models across folds was low, ranging from 0.12 to 0.22. Bayes B model had the least PA (*r =* 0.12) followed by Bayes C (*r =* 0.13), Bayesian Ridge Regression (*r =* 0.15), Bayesian Lasso (*r =* 0.16) and Bayes A (*r =* 0.19). Reproducing Kernel Hilbert Spaces model had higher PA (*r =* 0.22) than all the Bayesian models. All the Bayesian models did not perform differently (at α = 0.05) from each other (Fig. 8).

## Discussion and conclusions

Cassava is clonally propagated, has a long breeding cycle and complex genetics owing to its out crossing nature. Thus, genetic gains tend to be small compared to other crops [49–51]. Phenotyping for HCN trait is slow, laborious and may require specialized tools and/or personnel, making it restrictive for most resource constrained breeding programs. Moreover, it can only be done after the crop has attained physiological maturity, twelve months after planting. In contrast, genomic selection relies on genome-wide markers to predict the breeding values of individuals and has demonstrated significant potential, especially in early-stage breeding or in scenarios where phenotypic selection is difficult [52], such as with HCN. This study assessed the accuracy and predictive ability of seven genomic selection models—GBLUP, Bayes A, Bayes B, Bayes C, BL, BRR, and RKHS—for predicting the hydrogen cyanide (HCN) content in fresh cassava roots.

Phenotypic variation for HCN ranged from 1 to 9 in Namulonge and Serere and 4 to 9 in Ngetta. This shows that there was high variability for HCN within the study population. This is especially true given the fact that selection for low HCN clones had never been performed in this cyclic population. This variability can partly be explained by two possibilities. First, owing to the heterozygous nature of cassava and the two rounds of recombination to generate cycle 2 populations, it is anticipated that this population could have considerable standing genetic variation. Second, original founders for cycle 2 were sourced from diverse origins (i.e., in Africa and Latin America) and without significant positive selection for HCN. Thus, standing genetic variation was high as reflected in the PCA biplot (Fig. 4). This variability is suggestive of population stratification, which was accounted for using the kinship matrix. Estaghvirou et al. [36] demonstrated that genomic prediction accuracy in plant breeding increases with increase in genetic variation, especially when combined with increase in marker density and population size.

### Broad-sense heritability and narrow-sense heritability estimates

The broad-sense heritability estimate (H^2^ = 0.42) for the study was relatively low but still higher than the narrow-sense heritability (h^2^) estimates (h^2^ = 0.05–0.35). While narrow-sense heritability only considers additive genetic effects, broad-sense heritability captures the total genetic variation, including additive, non-additive, dominance, and epistatic effects [53]. The h^2^ was generally low, suggesting that non-additive effects may play a significant role in the genetic regulation of the trait. However, when using the BRR model, narrow-sense heritability (h^2^ = 0.66–0.70) surpassed broad-sense heritability, indicating the potential over estimation of additive variance. The BRR model assumes that all markers contribute small, normally distributed effects, thus applies uniform shrinkage [31]. However, since HCN is oligogenic, being controlled by a few large effect loci [20] it is possible that the model could have overestimated the effects of the small-effect loci and is thus over fitted.

### Population structure

Population structure, if unaccounted for can artificially inflate heritability estimates and genomic prediction accuracies as the model might capture family specific-signals rather than the true genetic signal [37, 54]. From the heatmap of the genomic relationship matrix (GRM) (Fig. 3B) and the PCA bi-plot (Fig. 4), it is clear that there was structure within the population, occasioned by relatedness among individuals from the same family. This clustering has practical implications for the breeding program. In order for the breeders to optimally utilize the diversity within this population, parents selected for the next cycle of genomic selection should be representative of individuals with superior GEBVs from the different clusters. The mating design selected should also maximize cross combinations between clusters.

To account for the evident population structure, in the GBLUP model, we incorporated the genomic relationship matrix (GRM). The GRM captures genetic similarities and differences between individuals based on SNPs and allows to adjust for any stratification (sub-populations that might have different allele frequencies) within the population by incorporating them into the genomic prediction model [55]. For the Bayesian models, population structure was accounted for using Markov chain Monte Carlo sampling which integrates population structure by sampling from the posterior distributions [56] while in the RKHS algorithm, population structure was accounted for by fitting the Gaussian kernel which can model complex genetic relationships and interactions within the population [57].

### Predictive accuracy and predictive ability of genomic models for HCN

Genomic predictive accuracy and predictive ability are two related but different concepts. Genomic prediction accuracy is the correlation between the genomic estimated breeding values (GEBVs) and the true breeding value divided by the square root of h^2^ [58, 59]. Accuracy is therefore a measure of how close predicted values (GEBVs) are to the actual HCN phenotype. Predictive ability (PA) on the other hand reflects how well a model can predict GEBVs for samples not included in the training set, making it a reliable indicator of the model's generalization capability [60].

In this study, PA values were consistently low across all models, ranging from 0.12 to 0.22. Among the models tested, RKHS and GBLUP achieved the highest PA, both reaching 0.22. This is comparable to the PA value (0.26) reported by Torres et al. [25] for HCN in a Nigerian cassava population, but much lower than that reported for Embrapa (0.59) and combined Nigerian and Embrapa populations (0.64) in the same study. For accuracy of genomic predictions, the GBLUP model has previously been demonstrated to be superior or perform as well as other more complex models [19, 28, 61, 62] for other traits while using less computational resources. The GBLUP model takes into consideration genetic relationships between individuals by considering the genomic relation matrix (GRM) constructed from SNP marker information. This contributes to increasing selection gains as covariance information amongst individuals is used to get GEBVs [61]. The prediction accuracy of GBLUP (*r =* 0.22) was lower than all other models except BRR (*r =* 0.18) and also lower than that reported by Torres et al. [25] for HCN using IITA-Nigerian germplasm (*r =* 0.51), Embrapa germplasm (*r =* 0.67) and the combined Embrapa and IITA population (*r =* 0.8–0.85). However, these predictive accuracies are comparable to accuracies reported for RKHS, Bayes A, Bayes B, Bayes C and BL (*r =* 0.48–0.52) by the same authors. The low predictive accuracy reported in this study could be attributed to the low broad-sense heritability of HCN (0.42) in the study population as compared to H^2^ of 0.76 and 0.56 reported for Embrapa and Embrapa-IITA populations [25]. The low H^2^ in this study suggests that a large proportion of the observed phenotypic variance in HCN is due non-additive genetic effects. Genomic prediction accuracy has been shown to increase with increase in trait heritability [40, 59].

The RKHS model which was more accurate than GBLUP with equal generalizing ability confers more advantages for selection. Reproducing Kernel Hilbert Spaces Regression was one of the earliest machine learning methods used in plant and animal breeding [63]. Kernel methods have gained wide acceptance due to their ability to effectively capture non-linear patterns in data, which linear statistical methods often fail to address [40]. This has been demonstrated in various studies, including those on cassava [28], chicken [64], and wheat [65, 66] despite the underlying differences in genetic architecture of the traits. Additionally, the RKHS algorithm is capable of accounting for both additive and non-additive effects [40]. However, some studies have found only minor differences between RKHS and linear methods [67, 68], reinforcing the idea that no single genomic prediction model is universally optimal for all datasets, as each model operates under different assumptions which may not hold true for certain traits. The use of multiple genomic prediction models combined into an ensemble has been demonstrated to improve genomic prediction accuracy and reduce prediction errors over individual genomic prediction models in a nested association mapping dataset of teosinte [69]. This as an approach that cassava breeding programs targeting low HCN varieties could also explore.

The superior performance of RKHS and Bayesian models to GBLUP could be attributed to the genetic architecture of HCN and the different assumptions made by the models on the gene effects. The genetic control of HCN is oligogenic, being mainly controlled by two genes on chromosome 14 and chromosome 16 [20]. Bayes A and B assume that marker effects are independent of each other, thus only a few markers are assumed to have large effects on the trait. Bayes C on the other hand assumes that only a small proportion of markers have moderate to large effects while BL assumes that most marker effects are small (zero) but a few may be large, thus unevenly shrinks markers, more aggressively shrinking small effect markers [31, 32]. All these model assumptions are congruent with the genetic architecture of HCN as an oligogenic trait. The superior predictive accuracy of RKHS can be attributed to its ability to model non-additive gene effects (dominance and epistasis) without having to explicitly specifying them in the model given its use of kernels [70]. Dominance and epistatic effects have both been reported for HCN [20]. On the other hand, the GBLUP assumes equal contribution (small effects) for all the markers across the genome [31, 40] thus fails to capture the enormous contribution of the large effect markers. An alternative to GBLUP would be the weighted GBLUP (WssBLUP) [38] in which markers can be assigned different weights based on their effects. For oligogenic HCN, the GWAS derived significant markers reported by Ogbonna et al. [20] can be incorporated as fixed effects into the GBLUP model such that the model can assign greater weight to these loci while still capturing background polygenic effects.

A major limitation in this study is that ordinal HCN scores were treated like Gaussian distributed quantitative data. For a model like the GBLUP that relies on portioning of genetic variance following Gaussian assumptions, the use of ordinal data was probably a mismatch between data type and model assumptions and could potentially have contributed to the low prediction accuracy reported with the GBLUP. The use of data generated from the modified picrate paper method which involves using the picrate paper in combination with a spectrophotometer [15] could therefore yield more compelling results.

### Practical implications for low cyanide cassava breeding

A recent study [20] reported that the genetic architecture of HCN is highly conserved and identified two major loci encoding for an ATPase and MATE protein, explaining up to 7% and 30% of the HCN phenotypic variation in the cassava root respectively. These loci were also shown to be in strong linkage disequilibrium (LD), which ensures that marker – trait associations are stable. The mate family proteins are involved in detoxification and are capable of transportation of cyanogenic glucosides [71]. This strong LD contributes to the prediction accuracies reported in this study since, proving that genomic prediction models can capture the genetic signal even when under the control of a few markers.

Although this study reports a low to moderate predictive accuracy for HCN, genomic selection could still significantly expedite the recurrent selection cycle by enabling the early selection of parents [22]. Besides recurrent selection, getting estimates of HCN (within limits) on unevaluated plots in multi-locational frameworks/sparse testing networks also enhances selection decisions. This approach could enhance genetic gains per unit of cost and time by eliminating poorly performing individuals, thereby reducing expenses related to field maintenance and phenotyping [72]. Furthermore, genomic selection-driven cyclic population improvement has the potential to swiftly increase the frequency of desirable alleles within a population. For instance, [73] demonstrated that conducting two genomic selection cycles over two years elevated the frequency of African cassava mosaic resistance alleles from 44 to 63%, achieving over twice the gains of traditional recurrent selection, with moderate genomic prediction accuracies of 0.53–0.58. Similarly, [74] observed 4 to 43% higher maize grain yield after two genomic selection cycles compared to pedigree-based selection in bi-parental populations, while [75] reported annual yield improvements of 1.2% to 2.8% in a multi-parental tropical maize population using genomic selection.

Genomic selection improves breeding program efficiency by allowing large populations to be screened early at minimal cost, which not only increases the selection intensity but also reduces the cycle time. For example, under traditional recurrent selection, HCN phenotypic evaluation and subsequent selection is done at crop maturity, which is 12 months after planting. Using genomic selection, genotyping can be done of one month-old seedlings and selection of parents done using GEBVs. This cuts the cycle by over 8 months. This, combined with its ability to increase selection intensity and accuracy presents genomic selection as a suitable vehicle to expeditiously deliver desired genetic gains for low HCN cassava varieties to farmers’ fields.

From the results presented, it is evident that choice of model has a big implication on the accuracy of genomic predictions for HCN. It was also apparent that increasing accuracy of the models resulted into improved predictive ability, making the models more reliable. Therefore, it pays for breeders to improve genomic prediction accuracy. Other important considerations for improving HCN prediction accuracy will be improving field experimental designs and optimizing laboratory HCN data collection protocols to minimize the error variance and thus increase trait heritability. Increasing the size of the training population, increasing marker density as well as including HCN trait associated markers are all options that could be explored [28, 38, 76]. The genomic prediction accuracies reported paint a picture of great potential. However, fresh cassava root HCN phenotypic expression is highly dependent on the environment [77]. Indeed, this study has also reported variability for HCN across test environments with Namulonge (mean HCN = 5.3) registering lower average scores compared to Ngetta (mean HCN = 6.4) and Serere (mean HCN = 6.6). In fact, HCN phenotypic expression is so variable that there are significant differences within roots of the same plant, even for different parts of the same root [78]. Thus, breeding programs embarking on HCN phenotyping must adopt standardized protocols for root sampling and processing [42]. Given this environment driven variability in HCN phenotypic expression, at the late stages of breeding when accession numbers are few (advanced yield trials or pre-commercial release stage), confirmatory laboratory based HCN phenotyping is necessary as a quality control step to genomic selection.

Given the phenotypic plasticity of fresh cassava root HCN content, it would be ideal for public sector cassava breeding programs to aim for low HCN varieties that are adapted to specific environments. However, given the chronic resource constraints, most programs opt to breed for wide adaptability by testing in representative target environments. To optimize resources, while deploying genomic selection, it would be ideal to use data of one environment to predict the performance of clones in another environment. However, when the genotype by environment signal is strong as is the case for HCN [77], the genetic signal captured in one environment may not be transferrable to another [79, 80]. Thus, the use of multi-environment data and genomic selection models that account for the genotype by environment interaction helps to improve the utility of the predictions. Ultimately, breeding for low HCN cassava varieties not only improves food safety but is also a gender responsive intervention that promises to relieve women of the burden of manual processing to detoxify cassava which disproportionately falls on them [81].

Although the cost of genotyping and thus conducting genomic selection has drastically reduced over the years [82], an estimated $21 per sample for high density genotyping with DArTseq [83] is still very high for publicly funded breeding programs especially given that they typically evaluate hundreds to thousands of accessions annually. Thus, there is need for cheaper and equally accurate or even more accurate methods for predicting performance and facilitating selection of desired individuals in large breeding programs [84] proposed the use of near infrared spectroscopy (NIRS) as a high throughput, low cost and non-destructive tool for indirectly capturing endophenotype variation and computing relationship matrices for predicting complex traits. This was termed as phenomic prediction (PP).

While GS relies on genome-wide markers to predict the phenotype of individuals, PP replaces molecular markers with NIRS spectra to deduce relationships among individuals and do selection, attaining results comparable to GS [85]. The SNP marker derived genomic relationship matrix is substituted with a NIRS spectra derived relationship matrix [85]. Since NIRS spectra reveal the biochemical composition of tissue, the variation observed in these spectra is linked to the chemical bonds within the sample tissue (endophenotypes). The principle behind NIRS aligns with the Beer-Lambert law [86], which states that absorbance or reflectance at a specific wavelength is directly proportional to the analyte's concentration. Reflectance at each wavelength represents an integration of various endophenotypic variations, meaning that a relationship matrix built from these spectra could potentially capture a genetic signal [84, 87]. Moreover, spectra could be used simultaneously with molecular markers to increase genomic prediction accuracies [88, 89].

In conclusion, our study has provided a critical evaluation of genomic prediction models for HCN in fresh cassava roots and demonstrated that genomic prediction can be effectively used to breed for low HCN cassava varieties. Whereas the commonly used GBLUP model showed low accuracy, the RKHS and the Bayesian Lasso model out performed all other models, achieving moderate accuracy. This highlights the importance of selecting models that capture both the additive and non-additive genetic effects when making genomic predictions for fresh cassava root HCN content. This work lays important groundwork for cassava breeding programs to integrate genomic selection for low HCN in their variety development pipeline, thus accelerating the rate of delivering desired genetic gains to farmers’ fields.

## Data Availability

Data sets generated and/or analysed are available from Cassavabase, an open access cassava breeding database via the link the [https://cassavabase.org/breeders/trial/6707?format=].
